# Effects of Exercise Training on Cardiopulmonary Function and Quality of Life in Elderly Patients with Pulmonary Fibrosis: A Meta-Analysis

**DOI:** 10.3390/ijerph18147643

**Published:** 2021-07-18

**Authors:** Xiaohan Li, Rongfang Yu, Ping Wang, Aiwen Wang, Huiming Huang

**Affiliations:** 1 Faculty of Sport Science, Research Academy of Grand Health, Ningbo University, Ningbo 315211, China; leexiaohan996@gmail.com; 2 School of Physical Education & Sport Training, Shanghai University of Sport, Shanghai 200438, China; yurongfang2009@126.com; 3 School of Physical Education and Exercise Sciences, Lingnan Normal University, Zhanjiang 524048, China; wp2923@163.com

**Keywords:** pulmonary fibrosis, exercise training, elderly health, pulmonary function, chronic diseases

## Abstract

(1) Objective: Our objective was to conduct a meta-analysis of randomized controlled trials that have evaluated the benefits of exercise training for elderly pulmonary fibrosis (PF) patients. (2) Methods: Studies in either English or Chinese were retrieved from the China National Knowledge Infrastructure (CNKI) and the Wanfang, PubMed, Web of Science and SPORTDiscus databases from inception until the first week of April 2021. Age, body mass index (BMI), and exercise frequency, intensity, type, and duration were considered for each participant. The specific data recorded were the six-minute walk distance (6MWD), maximal rate of oxygen consumption (peak VO_2_), predicted forced vital capacity (FVC% pred), predicted diffusing capacity of the lung for carbon monoxide (DLCO% pred), predicted total lung capacity (TLC% pred), St. George’s respiratory questionnaire (SGRQ) total score and a modified medical research council score (mMRC). (3) Results: Thirteen studies comprised this meta-analysis (eleven randomized controlled trials and two prospective studies design), wherein 335 patients were exercised and 334 were controls. The results showed that exercise training increased the 6MWD (Cohen’s *d* = 0.77, MD = 34.04 (95% CI, 26.50–41.58), *p* < 0.01), peak VO_2_ (Cohen’s *d* = 0.45, MD = 1.13 (95% CI, 0.45–1.82), *p* = 0.0001) and FVC% pred (Cohen’s *d* = 0.42, MD = 3.94 (95% CI, 0.91–6.96), *p* = 0.01). However, exercise training reduced scores for the SGRQ (Cohen’s *d* = 0.89, MD = −8.79 (95% CI, −10.37 to −7.21), *p* < 0.01) and the mMRC (Cohen’s *d* = 0.64, MD = −0.58 (95% CI, −0.79 to −0.36), *p* < 0.01). In contrast, exercise training could not increase DLCO% pred (Cohen’s *d* = 0.16, MD = 1.86 (95% CI, −0.37–4.09), *p* = 0.10) and TLC% pred (Cohen’s *d* = 0.02, MD = 0.07 (95% CI, −6.53–6.67), *p* = 0.98). Subgroup analysis showed significant differences in frequency, intensity, type, and age in the 6MWD results (*p* < 0.05), which were higher with low frequency, moderate intensity, aerobic–resistance–flexibility–breathing exercises and age ≤ 70. Meanwhile, the subgroup analysis showed significant differences in exercise intensity and types in the mMRC results (*p* < 0.05), which were lower with moderate intensity and aerobic–resistance exercises. (4) Conclusions: Exercise training during pulmonary rehabilitation can improved cardiopulmonary endurance and quality of life in elderly patients with PF. The 6MWDs were more noticeable with moderate exercise intensity, combined aerobic–resistance–flexibility–breathing exercises and in younger patients, which all were not affected by BMI levels or exercise durations. As to pulmonary function, exercise training can improve FVC% pred, but has no effect on DLCO% pred and TLC% pred.

## 1. Introduction

Pulmonary fibrosis (PF) is a devastating form of chronic, progressive, fibrosing interstitial pneumonia, which usually results from bacterial or viral infection, drugs, the environment, or disease [1]. Dyspnea is the cardinal symptom of PF that limits activity and impairs the quality of life of patients with PF [2]. PF usually occurs in the elderly over 70 years old. [3]. A recent study suggested that aging accompanies the increased PF likelihood [4]. Gaohong Sheng and colleagues suggested that a viral infection increases the risk of contracting PF [5]. In fact, PF is a severe post-infection complication of the respiratory disease COVID-19, caused by the SARS-CoV-2 virus [5,6,7,8]. Drug therapy is used to relieve the symptoms and delay the decline of lung function of patients with PF, but the overall efficacy is still unsatisfactory, and the cost and side-effects are not trivial [9,10,11,12]. Rehabilitation protocols with fewer side-effects for PF patients need to be explored.

Exercise training is conducive to sustaining health and has been widely reported for the prevention of and rehabilitation from chronic conditions [13]. Regular exercise training (e.g., swimming) was reported to improve pulmonary function in normal people [14]. Some interventional studies have suggested that exercise training decreases declines in pulmonary function. Vainshelboim’s study showed the enhancement in exercise capacity, dyspnea and quality of life among patients with PF after exercise interventions [15]. A study by Gaunaurd et al. showed that exercise training improved the level of physical activity of patients with PF and improved their quality of life [2]. Exercise training can be considered as a potential beneficial therapy for PF patients with improvements in six-minute walk distance (6MWD), dyspnea, quality of life and peak exercise capacity [16,17,18]. However, there are some contradictions in subsequent evaluations [2,19,20]. There are inconsistent reports that exercise training improves the life of PF patients in various aspects, including physical activity and pulmonary function. It has been suggested that patients with PF might benefit less from exercise training than patients with other disease etiologies [17,21]. Thus, the effectiveness on PF is less certain. Even though as assistant methods, exercise training, such as square dancing in the quarantine area, were encouraged for either young or elderly people during treatments in China by clinicians when curing the COVID-19 patients, it still needs to be determined whether exercise training can facilitate treatment in elderly PF patients.

This study aimed to combine the evidence from current studies to explore whether exercise training can improve cardiopulmonary fitness, pulmonary function and quality of life in elderly PF patients. Exercise training in this paper refers to activities associated with aerobic exercise, flexibility exercise, breathing exercise and resistance exercise. We expect to provide insights into pulmonary rehabilitation in elderly PF patients.

## 2. Materials and Methods

### 2.1. Registration

The protocol was prospectively registered on the PROSPERO International Prospective Register for Systematic Reviews website (Registration #: CRD42021224513) in December 2020. Design and reporting of this review have followed “Preferred Reporting Items for Systematic Reviews and Meta-Analyses” (PRISMA) statement.

### 2.2. Literature Search Strategy

Using guidelines provided by the Cochrane Collaboration, a comprehensive search strategy was devised and applied to the following electronic databases in the first week of April 2021 with no date restrictions: (1) China National Knowledge Infrastructure (CNKI) and the Wanfang, PubMed, Web of Science, SPORTDiscus databases. Articles published in English and Chinese were included, and all terms were searched as free text and keywords where applicable. Scientific databases were searched according to three criteria: participants (“pulmonary fibrosis patients”), medical interventions (“training or exercise”, “exercise training”, “pulmonary rehabilitation”, “physical exercise”, “exercise program” or “physical training”) and outcomes (“cardiopulmonary fitness or function”, “pulmonary function or lung function”, “quality of life”, “health-related quality of life”, “HRQL”, or ”QOL”). All search strategies were performed in English and Chinese in the relevant databases. All literature was imported into Endnote X9 (Thomson Reuters, Carlsbad, CA, USA), which also removed duplications. Two reviewers screened all titles and abstracts. Once abstracts suggested that studies were potentially suitable, the full-text versions were screened and then included in the review if they fulfilled the selection criteria. A third reviewer was consulted in cases of disagreements. Additional searches included reference list screening and citation tracking (Google Scholar) of all studies.

### 2.3. Selection Criteria

#### 2.3.1. Inclusion Criteria

(I) Studies included PF patients referring to the elderly, defined by the World Health Organization as those aged over 60 years old [22].

(II) Medical interventions related to the operating group consisting of aerobic, resistance, flexibility, and breathing exercises. For the control group, physical therapy and medication under the supervision of a therapist, playing Wii (a video game), and educational lectures.

(III) Studies that included any of the following criteria: six-minute walk distance (6MWD) [23], maximal rate of oxygen consumption (peak VO_2_), predicted forced vital capacity (FVC% pred), predicted diffusion capacity of the lung for carbon monoxide (DLCO% pred), predicted total lung capacity (TLC% pred), St. George’s respiratory questionnaire (SGRQ) [24], and a modified medical research council (mMRC) score [14]. An Egger test based on regression was used to analyze publication bias.

(IV) The study design was either a randomized controlled trial or a prospective study design.

#### 2.3.2. Exclusion Criteria

(I) Case reports;

(II) Non-English/Chinese study;

(III) Participants with an inventory of interpersonal problems (IIP), connective tissue disorders or extra parenchymal causes of restriction;

(IV) Cross sectional, retrospective, systematic reviews, editorial letters or conference abstracts without the full text available.

### 2.4. Reported Methodological Quality Assessment

Two independent reviewers rated the quality of studies using the Physiotherapy Evidence Database (PEDro) scale [25]. Any discrepancies were resolved by consensus, with a third reviewer available if needed. This PEDro scale consists of 11 items including eligibility criteria, random allocation, concealed allocation, baseline comparability, blinded subjects, blinded therapists, blinded assessors, adequate follow-up, intention to treat, between-group analysis, point measures and variability measures. The maximum score is out of 10 points, because item 1 only affects the external validity [25]. An excellent study had a PEDro score of 9 or 10, good (6–8), fair (4 or 5), and finally, poor (3 or lower). Of the studies, 11 were of good quality (GQ) [2,14,15,19,24,26,27,28,29,30,31] and two were fair quality (FQ) [32,33] (Table 1). Cut-off points for quality were determined following discussion among three people in the research team who had experience completing similar systematic reviews [34].

### 2.5. Data Management

Data (means and standard deviations (SD)) pertaining to participant and study characteristics were extracted and entered into an Excel spreadsheet.

#### 2.5.1. Outcomes

(I) Cardiopulmonary function: Within this review, cardiopulmonary function indexes included peak VO_2_, FVC% pred, DLCO% pred, and TLC% pred. Peak VO_2_ was used to predict cardiovascular disease in adults [34,35] and overall mortality [36,37]. The peak VO_2_ was measured by spiroergometry; specifically, exercising on a bicycle ergometer or treadmill until the subject reached their maximum. The single-breath diffusing capacity for carbon monoxide (DLCO) was also included. All values were expressed as a percentage of the predicted values reported. The FVC% pred was used to evaluate lung function, determining to what degree it had decreased. It is also useful for assessing the progression of lung disease and to evaluate the effectiveness of treatment [38]. The DLCO% pred was widely used in the diagnosis, classification, treatment, monitoring, and prognosis of PF patients [39].

(II) Quality of life: The six-minute walk test (6MWT) assessed functional limitations and determined functional capacity. As a self-paced and submaximal test, the 6MWD also reflects the ability to conduct daily activities [40,41]. The SGRQ is a disease-specific quality of life assessment tool validated for PF [42,43,44], where a high score implies a poor quality of life [45]. The mMRC scale is a self-rating tool to detect the degree to which breathlessness limits daily activity [46,47].

#### 2.5.2. Statistical Analysis

The effect size was calculated according to Cohen’s *d* [48]. Cohen suggested that *d* values of 0.2, 0.5, 0.8 represent small, medium, and large effect sizes, respectively [49]. This was calculated using Equations (1) and (2):

(1) $$d=\frac{MD_{1}-MD_{2}}{SD\left(pooled\right)}$$

(2) $$SD\left(pooled\right)=\sqrt{\frac{\left(S{D_{1}}^{2}+S{D_{2}}^{2}\right)}{2}}$$

The mean difference (*MD*) and *SD* were calculated using Equations (3) and (4):

(3) $$MD=MD_{1}-MD_{2}$$

(4) $$SD=\sqrt{S{D_{1}}^{2}+S{D_{2}}^{2}-2R\cdot SD_{1}\cdot SD_{2}}$$

The number 1 represents the baseline, and number 2 represents the follow-ups. We assumed an *R* value of 0.40 to impute the missing SD of the mean within-group change according to Follman et al. [50]. In this study, the effect size was represented by *d*, and the result size by *MD*. If a study reported results for different durations, each of them was treated as a separate trial [51]. The Cochrane systematic review software Review Manager (version 5.3.5) was used to map the forest. In addition, the 95% confidence intervals were calculated by this software. Meta-analysis was conducted to evaluate the effects of training interventions on PF. Heterogeneity in the studies was analyzed by a forest plot, and the heterogeneity was quantitatively determined by I^2^. This study had a low heterogeneity; therefore, a fixed-effect model was adopted for meta-analysis. If there was statistical heterogeneity among the results, its source was analyzed further, and significant heterogeneity was treated by subgroup analysis.

According to the characteristics of the studies, we conducted a subgroup analysis based on the exercise frequency, intensity, type, duration, age and body mass index (BMI). Exercise types were aerobic–resistance–flexibility–breathing exercise (A–R–F–B), aerobic–resistance exercise (A–R) and aerobic–flexibility exercise (A–F). Exercise frequency was divided into low (≤60 min × 2/week) and high (>60 min × 2/week) [52,53]. Exercise intensity was divided into light and moderate according to the American College of Sports Medicine (ACSM). ACSM define moderate intensity as 40% to 59% HRR or %VO_2_R, or 64% to 76% HRmax, or 46% to 63% VO_2_max, or fairly light to somewhat hard (RPE 12–13), or fairly light to somewhat hard (RPE 12–13), and light intensity was defined as 30% to 39% HRR or %VO_2_R, or 57% to 63% HRmax, or 37% to 45% VO_2_max, or very light–fairly light (RPE 9–11) [52]. Studies were divided into two groups, a group with age > 70 (71–95) and a group with age ≤ 70 (41–70) [54,55]. The BMI was divided into low (≤25) and high (>25) [56]. Exercise duration was divided into short (≤3 months) and long (>3 months) [19,26].

## 3. Results

### 3.1. Search Results and Reported Quality

**Figure 1:**
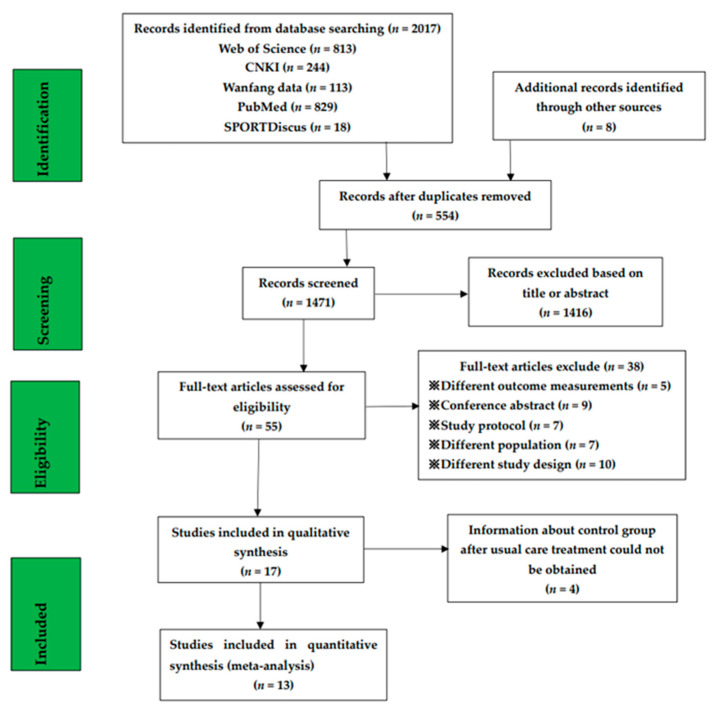
Flow chart of retrieval, screening and inclusion of articles in the systematic review.

Figure 1 shows the retrieved results. To the first week of April 2021, 2017 studies were confirmed from five databases and other sources in the search. After duplicate publications were deleted, 55 publications were evaluated by their titles. A total of 17 qualitative articles were obtained and 13 quantitative studies were confirmed to be included. No Chinese literature meeting the inclusion criteria was found. The main reasons for exclusion were outcome indicators that did not fulfill inclusion criteria. The selection process is shown in Figure 1. The characteristics of all the 13 included studies were based on frequency, intensity, duration, and type (FIDT) (Table 2). The operational and control groups of 13 studies were all PF patients [2,16,17,19,24,26,27,28,29,30,31,32,33]; thirteen studies compared interventions with exercise training to regular cares. Thirteen studies concerned aerobic exercise [2,16,17,19,24,26,27,28,29,30,31,32,33]. Twelve concerned resistance exercise [2,16,17,19,24,26,27,28,29,30,31,33]. Five studies included flexibility exercises [16,19,26,27,29] and four studies included breathing exercises [16,19,26,27]. Eleven studies had an exercise duration of three months or less [2,16,17,19,24,27,28,29,30,31,32], and five studies had a duration of more than three months [19,24,26,31,33]. The quality assessment results showed a decline in individual studies and black quality index scores, as shown in Table 1. Of the 13 included studies, eleven were of GQ [2,16,17,19,24,26,27,28,29,30,31] and two were FQ [32,33].

### 3.2. Cardiopulmonary Function

**Figure 2:**
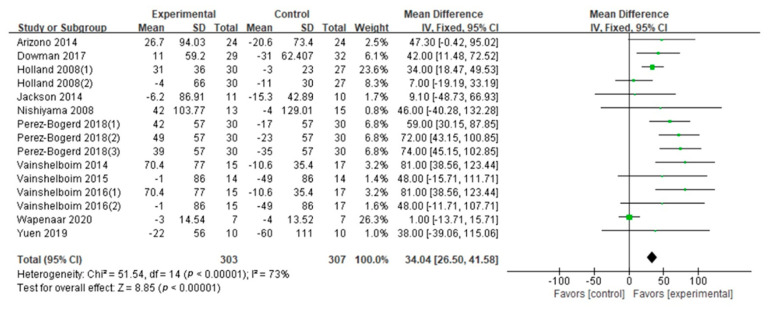
6MWD.

**Figure 3:**
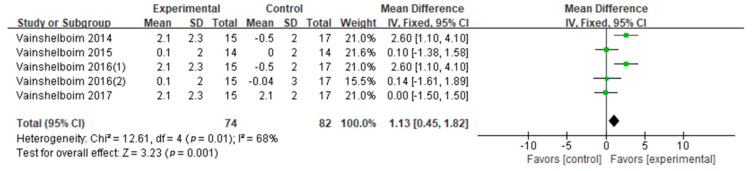
Peak VO_2_.

**Figure 4:**
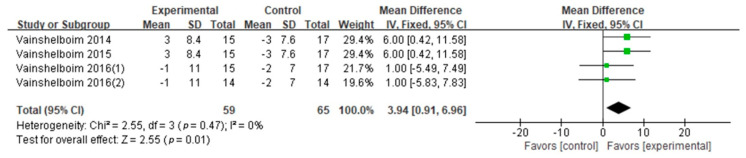
FVC% pred.

**Figure 5:**
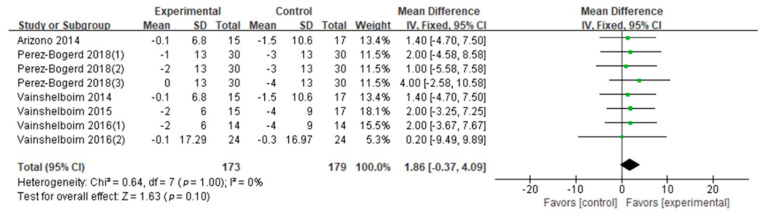
DLCO % pred.

**Figure 6:**
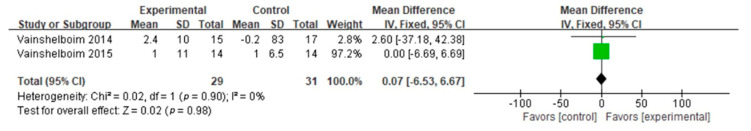
TLC% pred.

- 6MWD: The 6MWD was evaluated in eleven studies (nine GQ [16,17,19,24,26,28,29,30,31], two FQ [32,33]). The effect sizes were calculated according to Cohen’s *d*. The difference of the 6MWD between two conditions supported PF patients in exercise training (Cohen’s *d* = 0.77, MD: 34.04; 95% CI: 26.50 to 41.58; Z = 8.85, *p* < 0.01) (Figure 2). According to the information (exercise frequency, intensity, type, duration, age and BMI), we conducted the subgroup analysis (Table 3). This showed significant differences in frequency, intensity, type, and age on the 6MWD results (Table 3). Compared with the high frequency group (>60 min × 2/week; Cohen’ *d* = 0.82, *p* = 0.001), the 6MWD in the low frequency group (≤60 min × 2; Cohen’s *d* = 0.62) was higher. The 6MWD was higher at moderate exercise intensity (Cohen’s *d* = 0.78) than at light exercise intensity (Cohen’s *d* = 0.77, *p* = 0.004). Seven studies evaluated aerobic–resistance exercise (five GQ [17,24,28,30,31], two FQ [32,33]; Cohen’s *d* = 0.77, *p* < 0.01); one GQ study [29] evaluated aerobic–flexibility exercise (Cohen’s *d* = 0.13, *p* = 0.76); and three GQ studies [16,19,26] evaluated aerobic–resistance–flexibility–breathing exercise (Cohen’s *d* = 0.92, *p* < 0.01). Combined aerobic–resistance–flexibility–breathing exercise produced higher 6MWD (*p* = 0.0008). Three GQ studies [19,26,30] evaluated a long duration (Cohen’s *d* = 0.77, *p* < 0.01), and ten studies (eight GQ [16,17,19,24,28,29,30,31], two FQ [32,33]) evaluated a short duration (Cohen’s *d* = 0.78, *p* < 0.01). Although the exercise duration difference between subgroups was significant (*p* = 0.04), the confidence intervals overlapped. Hence, we concluded that there was no difference between exercise duration subgroups. Four GQ studies [17,29,31,33] evaluated subjects older than 70 (Cohen’s *d* = 0.45, *p* = 0.14), seven studies (six GQ [16,19,24,26,28,30], one FQ [32]) evaluated those 70 or younger (Cohen’s *d* = 0.86, *p* < 0.01). The 6MWD differences between the age subgroups (*p* < 0.01) suggested that the group aged under 70 derived more benefits from exercise training. Pertaining to BMI, five studies (four GQ [16,19,26,30], one FQ [33]) evaluated high BMI groups (Cohen’s *d* = 1.03, *p* < 0.01) and one GQ study [28] evaluated a low BMI group (Cohen’s *d* = 0.38, *p* = 0.30). Differences were not observed between two BMI levels (*p* = 0.62).
- Peak VO_2_: Peak VO_2_ was evaluated in four GQ studies [16,19,26,27]. The difference of the peak VO_2_ between two conditions supported PF patients engaging in exercise training (Cohen’s *d* = 0.45, MD: 1.13; 95% CI: 0.45 to 1.82; Z = 3.23, *p* = 0.0001) (Figure 3). Due to the high heterogeneity (I^2^ = 68%), a subgroup analysis of exercise duration was performed (Table 3). It that showed two GQ studies [26,27] focused on long durations (Cohen’s *d* = 0.74, *p* = 0.84) and three GQ studies [16,27,29] focused on short durations (Cohen’s *d* = 0.05, *p* < 0.01). Although the exercise duration difference between subgroups was significant (*p* = 0.03), the confidence intervals overlapped. Accordingly, we conclude that exercise duration has no effect on peak VO_2_.
- FVC% pred: FVC% pred was evaluated in three GQ studies [16,19,26]. The synthesized FVC% pred encouraged patients with PF to engage in exercise training (Cohen’s *d* = 0.42, MD: 3.94; 95% CI: 0.91 to 6.96; Z = 2.55, *p* = 0.01) (Figure 4). No difference was observed when we compared two exercise duration subgroups (*p* = 0.35) (Table 3).
- DLCO% pred: Five studies were included in the meta-analysis to provide DLCO% pred numerical data (four GQ [16,19,26,30], one FQ [32]). The combined DLCO% pred did not support patients with PF engaging in exercise training (Cohen’s *d* = 0.16, MD: 1.86; 95% CI: −0.37 to 4.09; Z = 1.63, *p* = 0.10) (Figure 5). Exercise frequency, duration, intensity and type subgroups had no significant difference in DLCO% pred results (*p* = 0.93, 0.84, 0.86, 0.86) (Table 3).
- TLC% pred: TLC% pred was evaluated in two GQ studies [16,26]. The difference of the TLC% pred between two conditions did not support patients with PF engaging in exercise training (Cohen’s *d* = 0.02, MD: 0.07; 95% CI: −6.53 to 6.67; Z = 0.02, *p* = 0.98) (Figure 6). There was no significant difference between the two exercise duration subgroups (*p* = 0.90) (Table 3).

### 3.3. Quality of Life

**Figure 7:**
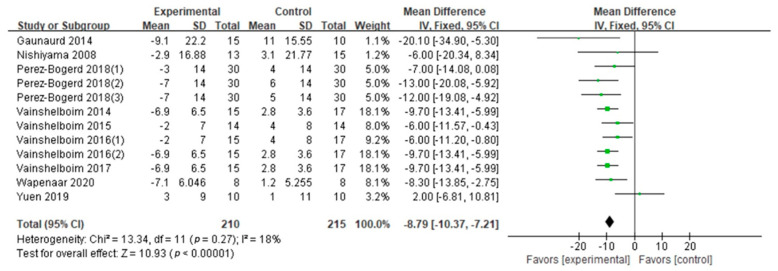
SGRQ.

**Figure 8:**
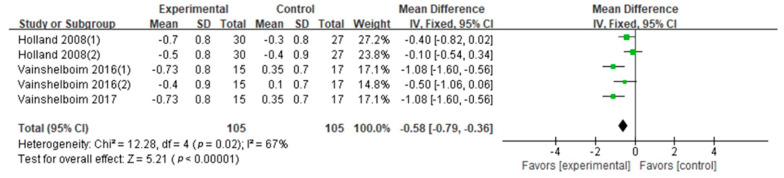
mMRC. Notes: Symbols: for single studies, the squares indicate the mean difference, and the relative size of the square is an indication of the weighting of this study towards the overall effect. The endpoints of the horizontal lines are the upper and lower 95% confidence intervals. The large diamonds represent the summed data for the subgroups and all studies included in the meta-analysis; the midpoint of the diamond indicates the mean difference, whereas the endpoints are the upper and lower 95% confidence intervals. Abbreviations: 95% CI, 95 percent confidence interval; IV, inverse variance; SD, standard deviation. If an included study reported results for different durations, each different duration was treated as a separate trial [51]. Vainshelboim 2016 (1) represents a study with a 3-month exercise duration. Vainshelboim 2016 (2) represents a study with an 11-month exercise duration. Holland 2008 (1) represents a study with a 2.25-month exercise duration. Holland 2008 (2) represents a study with a 6.5-month exercise duration. Perez-Bogerd 2018 (1) represents a study with 3-month exercise duration. Perez-Bogerd 2018 (2) represents a study with a 6-month exercise duration. Perez-Bogerd 2018 (3) represents a study with a 12-month exercise duration.

- SGRQ: SGRQ was evaluated in nine studies (eight GQ [2,16,19,26,27,28,30,31], one FQ [33]). The synthesized SGRQ in this study encouraged patients with PF to engage in exercise training (Cohen’s *d* = 0.89, MD: −8.79; 95% CI: −10.37 to −7.21; Z = 10.93, *p* < 0.01) (Figure 7). According to the relevant information (exercise frequency, intensity, type, duration, age and BMI), we conducted the subgroup analysis (Table 3). The results showed that SGRQ scores were not affected by exercise frequency, intensity, duration, type, age or BMI. Five studies (four GQ [28,29,30,31], one FQ [33]) evaluated aerobic–resistance exercise (Cohen’s *d* = 0.67, *p* = 0.0005), and four GQ studies [16,19,26,27] evaluated aerobic–resistance–flexibility–breathing exercise (Cohen’s *d* = 1.35, *p* < 0.01). There were no differences in exercise type between subgroups (*p* = 0.97). Two GQ studies [19,26] evaluated long duration (Cohen’s *d* = 1.00, *p* < 0.01), eight studies (seven GQ [2,16,19,27,28,30,31], one FQ [33]) evaluated short durations (Cohen’s *d* = 0.82, *p* < 0.01). There were no differences in exercise duration between subgroups (*p* = 0.43). Three studies (two GQ [2,31], one FQ [33]) evaluated subjects older than 70 (Cohen’s *d* = 0.63, *p* = 0.003); six GQ studies [16,19,26,27,28,30] evaluated those 70 or younger (Cohen’s *d* = 0.94, *p* < 0.01). There were no differences in age between subgroups (*p* = 0.33). Six studies (five GQ [16,19,26,27,30], one FQ [33]) evaluated high BMI (Cohen’s *d* = 1.00, *p* < 0.01), and one GQ study [28] evaluated low BMI (Cohen’s *d* = 0.30, *p* = 0.41). There were no differences in BMI between subgroups (*p* = 0.71).
- mMRC: mMRC was evaluated in three GQ studies [19,24,27]. The difference in the mMRC between two conditions supported patients with PF engaging in exercise training (Cohen’s *d* = 0.64, MD: −0.58; 95% CI: −0.79 to −0.36; Z = 5.21, *p* < 0.01) (Figure 8). Due to the high heterogeneity (I^2^ = 67%), subgroup analysis on exercise intensity, type and duration was performed (Table 3). The subgroup analysis showed significant differences in intensity and type on the mMRC. Although the exercise duration difference between subgroups was significant (*p* = 0.02), the confidence intervals overlapped. Hence, we conclude that there is no difference between exercise duration subgroups. The mMRC was higher at light exercise intensity (Cohen’s *d* = 1.11) and aerobic–resistance–flexibility–breathing exercise (Cohen’s *d* = 1.11) than at moderate exercise intensity (Cohen’s *d* = 0.30) and aerobic–resistance exercise (Cohen’s *d* = 0.30, *p* = 0.004).

### 3.4. Publication Bias

For all studies, the potential publication bias was evaluated by Egger’ regression test [57]. Egger’ regression tests were performed for 6MWD, Peak VO_2_, FVC% pred, DLCO% pred, TLC% pred, SGRQ and mMRC. The *p*-values were all greater than 0.05 (*p* > 0.05), suggesting no publication bias.

## 4. Discussion

The results in this study indicated that exercise training could improve cardiopulmonary endurance and the quality of life.

### 4.1. Cardiopulmonary Function

#### 4.1.1. Cardiopulmonary Endurance

In this study, cardiopulmonary endurance was evaluated with peak VO_2_, which is widely used to assess cardiopulmonary endurance by researchers [58]. In our study, a medium effect size was found, which indicated that exercise training improve peak VO_2_ performance in PF patients. Due to the high heterogeneity, subgroup analysis on exercise duration was performed. The confidence intervals of the exercise duration subgroups overlapped, and the effect size was very small when exercise duration was less than or equal to 3 months (Table 3); therefore, the peak VO_2_ might not be affected by exercise duration. Exercise types in older adults should include aerobic, resistance, flexibility, balance training, etc. [59]. Our results further indicate that combined aerobic–resistance–flexibility–breathing training can improve the cardiopulmonary endurance of elderly patients with PF.

The findings in the current study support the previous hypothesis that exercise training improves cardiopulmonary endurance in patients with PF [39]. One GQ study showed that exercise training significantly improved peak VO_2_ in the elderly in both healthy and disease contexts [60]. Exercise training (2–3 times per week) can effectively improve joints’ range of motion and muscle endurance [52]. Especially in the elderly, exercise training preserves bone mass and reduces the risk of falling [61]. The increase in peak VO_2_ in the operate group [62] is presumably because long-term exercise training increases cardiopulmonary endurance through improving blood circulation, lowers blood pressure, and improves cardiovascular function [62]. Therefore, the current synthesized evidence supports the opinion that exercise training can improve the cardiopulmonary endurance of PF patients.

#### 4.1.2. Pulmonary Function

Pulmonary function was evaluated with the FVC% pred, DLCO% pred and TLC% pred in this study. One study showed that the loss of pulmonary function may lead to ventilatory limitation in exercise training in the active elderly, which decreases the accumulation of health benefits during physical activity [63]. FVC% pred can be used as an indicator of disease progression, which can be combined with other variables to predict disease progression more accurately [64]. PF patients expand their lungs with more difficulty due to a narrower airway [65]; one study revealed that exercise training can expand airways to increase FVC% pred in healthy subjects [66]. This may provide insights for PF patients.

The results in the current study indicate that exercise training improves FVC% pred performance in patients with PF. Disease progression in PF is monitored by a decline in forced vital capacity (FVC). An absolute or relative decline in FVC% pred of ≥10% is associated with mortality [67,68,69]. Two of the GQ studies on FVC% pred supported exercise training [16,26], whereas two other GQ studies did not support it [19,27]. The combined evidence supported the positive effect of exercise training on FVC% pred. However, the limitations were that data were extracted from one author and all studies were from the same group. Therefore, the effect of exercise training on FVC% pred is still inconclusive and needs to be further studied.

DLCO% pred provided an objective index of disease severity and prognosis [70], which is related to the rate of oxygen uptake by hemoglobin [71]. This study showed that exercise frequency, intensity, type, and duration did not affect the DLCO% pred. The number of studies on the DLCO% pred was abundant and the pool of subjects was large (*n* = 8, *n* = 98, respectively); therefore, the lack of benefit from exercise training on DLCO% pred of PF patients was validated.

Only two GQ studies focusing on the effect of aerobic–resistance–flexibility–breathing exercise reported TLC% pred; the results showed that aerobic–resistance–flexibility–breathing exercise had no benefits on the TLC% pred. No study with an adequate sample size (*n* ≥ 30) was found to evaluate the effects of exercise training on TLC% pred. The effect of exercise training on TLC% pred in patients with PF needs further study. Compared with other physical therapy methods, exercise training has merely no side-effects on patients with PF; thus, patients will have a higher tolerance to exercise training [72]. However, prospective evidence is still needed.

### 4.2. Quality of Life

Quality of life was evaluated with 6MWD, SGRQ and mMRC in our study. Our results showed that exercise training improved 6MWD performance in PF patients. We further explored the effects of exercise frequency, intensity, duration, age and BMI on 6MWD (Table 3). The subgroups analysis showed that there were no differences in 6WMD between two BMI levels and two exercise duration groups, whereas there were differences among different exercise frequency, intensity, type and age groups. The differences between age groups can be supported indirectly by a recent study [4]. Our findings indicated that the effects of 6MWD are more obvious in moderate exercise intensity, combined exercise of four types and with younger patients; meanwhile, the effects were not affected by BMI level or exercise duration. In this study, the combined effect size of 6MWD was medium (Cohen’s *d* = 0.77). Therefore, elderly patients with PF can derive benefits from exercise training on 6WMD.

In this study, exercise training reduced SGRQ performance in PF patients (Figure 7). The SGRQ is a disease-specific quality of life assessment tool validated for both chronic obstructive pulmonary disease (COPD) and PF [42,43,44]. There were 76 items in the questionnaire, including three parts to measure symptoms, activity restriction and the social and emotional impact of the disease. A higher score implies a poorer quality of life [45]. Compared with the control group, the operational group scored lower on the SGRQ. Due to high heterogeneity, based on the included information (exercise frequency, intensity, type, duration, age and BMI), we conducted subgroup analysis. The results showed that no SGRQ differences could be found in frequency, intensity, type, duration, age or BMI groups. Thus, the SGRQ was probably not affected by frequency, intensity, type, duration, age or BMI. The effect of BMI on the SGRQ is still not clear, because there was one study which did not include BMI, and there were a few studies on BMI greater than 25.

Figure 8 shows a decreased mMRC score in the operational group compared with the control group. The mMRC scale is a self-rating tool to measure the degree of disability that breathlessness poses on daily activities [46,47]. The higher the score, the more severe the disability. Subgroup analysis showed that mMRC was not affected by exercise duration. Limited by the small pool of subjects, the findings were still inconclusive. In the future, the effects of exercise frequency, age and BMI on mMRC in PF patients need to be focused.

Overall, a large effect size suggests that exercise training reduces SGRQ performance, and medium effect size indicates that exercise training increase 6MWD performance. Improvement in the 6MWD equates with an improved quality of life in patients [73]. Additionally, a large effect size from this review indicated that exercise training had a positively impact on the mMRC. The PF patients were more breathless and tended to be less physically active [74,75]; consequently, their functional capacity and quality of life became worse [74,75,76]. Through exercise training during pulmonary rehabilitation, PF patients achieved an improvement in exercise ability and ventilation function, which alleviated dyspnea during sub-maximal exercises such as activities of daily living [52], the fact of which was demonstrated by a decrease in the mMRC after the exercise intervention. An active exercise training lifestyle can improve the quality of life by increasing feelings of vitality [77], well-being [78,79], and reduce the risk of cognitive decline and dementia [80,81,82,83]. Therefore, the comprehensive evidence in current study reveals that exercise training can improve the quality of life of patients with PF.

### 4.3. Advantages and Future Directions

In summary, this review has evaluated the effects of aerobic, resistance, flexible, and breathing exercise on cardiopulmonary endurance, pulmonary function, and quality of life in PF patients. However, compelling studies are still lacking in evaluating FVC% pred, TLC% pred and mMRC; therefore, more studies are needed in the future, especially on single interventions.

To the best of our knowledge, this meta-analysis has two advantages. Firstly, the data extraction was more reasonable and standard than other reviews due to comprehensive literature retrieval strategies. We searched for studies from five countries, three continents, and in two languages (English/Chinese), which further reduced regional bias and language bias. Secondly, we analyzed two methods of exercise training effects (Cohen’s *d*, and mean difference) which evaluated clinical and statistical effects.

The main limitations of this study were that the disease severity, variability and progression of the PF patients included were varied, which may have affected the results. Another limitation was that the included exercise training regimens were combined; thus, a single exercise type could not be evaluated.

## 5. Conclusions

Exercise training during pulmonary rehabilitation can improve cardiopulmonary endurance and quality of life in elderly patients with PF. The 6MWD were more noticeable with moderate exercise intensity, combined aerobic–resistance–flexibility–breathing exercises and in younger patients, all which were not affected by BMI levels or exercise durations. Regarding pulmonary function, exercise training can improve FVC% pred, but has no effect on DLCO% pred and TLC% pred.

## Figures and Tables

**Table 1:** PEDro (Physiotherapy Evidence Database) scale quality assessment.

	1	2	3	4	5	6	7	8	9	10	11	Score	Quality
Vainshelboim, 2014	Yes	1	1	1	0	0	0	1	1	1	1	7	good
Vainshelboim, 2015	Yes	1	1	1	0	0	0	1	1	1	1	7	good
Vainshelboim, 2016	Yes	1	1	1	0	0	0	1	1	1	1	7	good
Vainshelboim, 2017	Yes	1	1	1	0	0	0	1	1	1	1	7	good
Perez-Bogerd, 2018	Yes	1	1	1	0	0	0	1	1	1	1	7	good
Nishiyama, 2008	Yes	1	1	1	0	0	0	1	1	1	1	7	good
Yuen, 2019	Yes	1	0	1	0	1	0	0	0	1	1	5	fair
Jackson, 2014	Yes	1	1	1	0	0	0	1	1	1	1	7	good
Gaunaurd 2014	Yes	1	1	1	0	0	0	1	1	1	1	7	good
Dowman 2017	Yes	1	1	1	1	0	0	1	1	1	1	8	good
Holland, 2008	Yes	1	1	1	1	0	0	1	1	1	1	8	good
Arizono, 2014	Yes	1	0	1	0	0	0	1	0	1	1	5	fair
Wapenaar, 2020	Yes	1	1	1	0	0	0	1	1	1	1	7	good

Notes: 1 = eligibility criteria; 2 = randomization of the sample; 3 = concealed allocation; 4 = initial comparability between groups; 5 = all subjects were blinded; 6 = all therapists who administered therapy were blinded; 7 = all evaluators measuring key outcomes were blinded; 8 = adequacy of follow-up; 9 = analysis with intention to treat; 10 = statistical comparison of results between groups; 11 = existence of specific measures and variability for at least one key result. All questions were scored on the following scale: yes—1, unable to determine—0, no—0. Excellent quality = 9–10; good quality = 6–8; fair quality = 4–5; poor quality = <4.

**Table 2:** Characteristics of the included studies.

Study	Country Study Design	Sample Size (*n*)	Age (Year)	Male (%)	BMI (kg/m^2^)	Frequency (/Week)	Intensity	Duration (Months)	Type	Outcomes
Vainshelboim, 2014 [16]	Israel RCT	OG: 15 CG: 17	OG: 68.8 (6) CG: 66 (9)	OG: 67% CG: 65%	OG: 28.3 (3.5) CG: 28.8 (3.5)	60 min × 2	Light	3	OG: A–R–F–B CG: RC	①②③ ④⑤⑥
Vainshelboim, 2015 [26]	Israel RCT	OG: 14 CG: 14	OG: 68.8 (6) CG: 66 (9)	OG: 67% CG: 65%	OG: 28.3 (3.5) CG: 28.8 (3.5)	60 min × 2	Light	11	OG: A–R–F–B CG: RC	①②③ ④⑤⑥
Vainshelboim, 2016 [19]	Israel RCT	OG: 15 CG: 17	OG: 68.8 (6) CG: 66 (9)	OG: 67% CG: 65%	OG: 28.3 (3.5) CG: 28.8 (3.5)	60 min × 2	Light	3, 11	OG: A–R–F–B CG: RC	①②③ ④⑥⑦
Vainshelboim, 2017 [27]	Israel RCT	OG: 15 CG: 17	OG: 68.8 (6) CG: 66 (9)	OG: 67% CG: 65%	OG: 28.3 (3.5) CG: 28.8 (3.5)	60 min × 2	Light	3	OG: A–R–F–B CG: RC	②⑥⑦
Perez-Bogerd, 2018 [30]	Belgium RCT	OG: 30 CG: 30	OG: 64 (13) CG: 64 (8)	OG: 73% CG: 50%	OG: 28 (4) CG: 26 (5)	3 (1–3 months) 2 (4–12 months)	Moderate	3, 6, 12	OG: A–R CG: RC	①④⑥
Nishiyama, 2008 [28]	Japan RCT	OG: 13 CG: 15	OG: 68.1 (8.9) CG: 64.5 (9.1)	OG: 92% CG: 60%	OG: 23 (3.8) CG: 22.9 (2.8)	2	Moderate	2.5	OG: A–R CG: RC	①⑥
Yuen 2019 [33]	America RCT	OG: 10 CG: 10	OG: 67.4 (7.4) CG: 72.2 (8.4)	OG: 50% CG: 80%	OG: 28.0 (4.6) CG: 28.4 (4.3)	30 min × 3	Light	3	OG: A–R CG: RC	①⑥
Jackson, 2014 [29]	America RCT	OG: 11 CG: 10	OG: 71 (6) CG: 66 (7)	NC	NC	120 min × 2	Moderate	3	OG: A–F CG: RC	①
Gaunaurd, 2014 [2]	America RCT	OG: 11 CG: 10	OG: 71 (6) CG: 66 (7)	NC	NC	90 min × 2	Moderate	3	OG: A–R CG: RC	⑥
Dowman, 2017 [17]	Australia RCT	OG: 32 CG: 29	OG: 70 (10) CG: 73 (9)	OG: 66% CG: 69%	NC	2	Moderate	2.25	OG: A–R CG: RC	①
Holland, 2008 [24]	Australia RCT	OG: 30 CG: 27	OG: 70 (8) CG: 67 (13)	NC	NC	2	Moderate	2.25, 6.5	OG: A–R CG: RC	①⑦
Arizono, 2014 [32]	Japan Pre-post	OG: 24 CG: 24	OG: 69 (7) CG: 69 (6)	OG: 67% CG: 67%	NC	90 min × 2	Moderate	2.5	OG: A–R CG: RC	①④
Wapenaar, 2020 [31]	Netherlands Pre-post	OG: 10 CG: 10	OG: 71 (7) CG: 71 (7)	OG: 80% CG: 80%	NC	60 min × 6	Light	2	OG: A–R CG: RC	①⑥

Notes: Mean ± standard deviation (SD) unless otherwise specified. BMI: body mass index, RCT: randomized controlled trial, Pre-post: prospective study design, OG: operational group, CG: control group, A–R–F–B: aerobic–resistance–flexibility–breathing exercise, A–R: aerobic–resistance exercise, A–F: aerobic–flexibility exercise, RC: regular care, NC: unrecorded, moderate intensity: 40–59% HRR or %VO_2_R, or 64–76% HRmax, or 46–63% VO_2_max, or fairly light to somewhat hard (RPE 12–13), light intensity: 30–39% HRR or %VO_2_R, or 57–63% HRmax, or 37–45% VO_2_max, or very light–fairly light (RPE 9–11), ①: six-minute walk distance (6MWD), ②: maximal rate of oxygen consumption (peak VO_2_), ③: predicted forced vital capacity (FVC% pred), ④: predicted diffusing capacity of the lung for carbon monoxide (DLCO% pred), ⑤: predicted total lung capacity (TLC% pred), ⑥: St. George’s Respiratory Questionnaire total score (SGRQ), ⑦: modified Medical Research Council score (mMRC).

**Table 3:** Subgroup analysis results.

	Group Standard	Study Quantity	Sample Size (*n*)	Mean Difference (95% CI)	Cohen’s *d*	Z	I^2^	*p* ^a^	*p* ^b^
6MWD					0.77				
Frequency	>60 min × 2	4	OG:75/CG:74	15.09 (2.74, 27.43)	0.62	2.40	79%	0.02	0.0001
	≤60 min × 2	11	OG:234/CG:233	45.32 (35.80, 54.85)	0.82	9.33	56%	<0.00001	
Intensity	Light	6	OG:79/CG:85	19.64 (7.17, 32.11)	0.77	3.09	79%	0.002	0.004
	Moderate	9	OG:230/CG:222	42.34 (32.87, 51.81)	0.78	8.76	59%	<0.00001	
Type	A–R	10	OG:239/CG:232	30.71 (22.72, 38.71)	0.77	7.53	78%	<0.00001	0.0008
	A–F	1	OG:11/CG:10	9.10 (−48.73, 66.93)	0.13	0.31		0.76	
	A–R–F–B	4	OG:74/CG:82	70.38 (45.66, 95.10)	0.92	5.58	0%	<0.00001	
Duration	>3 months	5	OG:119/CG:118	48.07 (32.98, 63.17)	0.77	6.24	74%	<0.00001	0.04
	≤3 months	10	OG:190/CG:189	29.37 (20.67, 38.08)	0.78	6.61	72%	<0.00001	
Age	>70	4	OG:58/CG:51	9.54 (−3.19, 22.28)	0.45	1.47	51%	0.14	<0.00001
	≤70	11	OG:246/CG:248	47.26 (37.90, 56.62)	0.86	9.90	57%	<0.00001	
BMI	>25	8	OG:159/CG:165	68.01 (54.41, 81.60)	1.03	9.8	0%	<0.00001	0.62
	≤25	1	OG:13/CG:15	46.00 (−40.28, 132.28)	0.38	1.04		0.3	
Peak VO_2_					0.45				
Duration	>3 months	2	OG:29/CG:31	0.12 (−1.01, 1.25)	0.74	0.2	0	0.84	0.03
	≤3 months	3	OG:45/CG:51	1.73 (0.87, 2.60)	0.05	3.92	74%	<0.00001	
FVC% pred					0.42				
Duration	>3 months	1	OG:15/CG:17	1.00 (−5.83, 7.83)	0.11	0.29		0.77	0.35
	≤3 months	3	OG:94/CG:98	4.65 (1.28, 8.02)	0.51	2.70	0%	0.007	
DLCO% pred					0.16				
Frequency	>60 min × 2	2	OG:54/CG:54	1.68 (−2.80, 6.15)	0.15	0.73	0%	0.46	0.93
	≤60 min × 2	6	OG:119/CG:125	1.92 (−0.65, 4.49)	0.17	1.46	0%	0.14	
Intensity	Light	4	OG:59/CG:65	1.66 (−1.42, 4.75)	0.15	1.06	0%	0.29	0.86
	Moderate	4	OG:114/CG:114	2.07 (−1.15, 5.30)	0.17	1.26	0%	0.21	
Type	A–R	4	OG:114/CG:114	2.07 (−1.15, 5.30)	0.17	1.26	0%	0.21	0.86
	A–R–F–B	4	OG:59/CG:65	1.66 (−1.42, 4.75)	0.15	1.06	0%	0.29	
Duration	>3 months	3	OG:75/CG:77	1.27 (−2.94, 5.47)	0.11	0.59	0%	0.55	0.84
	≤3 months	5	OG:98/CG:102	1.78 (−0.86, 4.41)	0.18	1.32	0%	0.19	
TLC% pred					0.02				
Duration	>3 months	1	OG:14/CG:14	0.00 (−6.69, 6.69)	0.00	0.00		1.00	0.90
	≤3 months	1	OG:15/CG:17	2.60 (−37.18, 42.28)	0.04	0.13		0.90	
SGRQ					0.89				
Frequency	>60 min × 2	2	OG:40/CG:40	−7.81 (−12.18, −3.44)	0.65	3.50	0%	0.0005	0.64
	≤60 min × 2	10	OG:168/CG:177	−8.94 (−10.63, −7.25)	0.95	10.36	31%	<0.00001	
Intensity	Light	7	OG:94/CG:102	−8.34 (−10.07, −6.61)	1.15	9.43	28%	<0.00001	0.22
	Moderate	5	OG:114/CG:115	−10.96 (−14.76, −7.16)	0.71	5.65	0%	<0.00001	
Type	A–R	7	OG:134/CG:135	−8.75 (−11.70, −5.79)	0.67	5.80	43%	<0.00001	0.97
	A–R–F–B	5	OG:74/CG:82	−8.81 (−10.67, −6.94)	1.35	9.26	0%	<0.00001	
Duration	>3 months	4	OG:104/CG:108	−9.65 (−12.27, −7.02)	1.00	7.2	0%	<0.00001	0.43
	≤3 months	8	OG:104/CG:109	−8.31 (−10.28, −6.34)	0.82	8.26	28%	<0.00001	
Age	>70	3	OG:31/CG:30	−6.72 (−11.20, −2.24)	0.63	2.94	72%	0.003	0.33
	≤70	9	OG:177/CG:187	−9.09 (−10.77, −7.40)	0.94	10.57	0%	<0.00001	
BMI	>25	9	OG:174/CG:182	−8.73 (−10.40, −7.06)	1.00	10.27	27%	<0.00001	0.71
	≤25	1	OG:13/CG:15	−6.00 (−20.34, 8.34)	0.30	0.82		0.41	
mMRC					0.64				
Intensity	Light	3	OG:45/CG:54	−0.91 (−1.21, −0.60)	1.11	5.73	30%	<0.00001	0.004
	Moderate	2	OG:60/CG:54	−0.26 (−0.56, 0.04)	0.30	1.68	0%	0.09	
Type	A–R	2	OG:60/CG:54	−0.26 (−0.56, 0.04)	0.30	1.68	0%	0.09	0.004
	A–R–F–B	3	OG:45/CG:54	−0.91 (−1.21, −0.60)	1.11	5.73	30%	<0.00001	
Duration	>3 months	2	OG:45/CG:44	−0.25 (−0.60, 0.10)	0.29	1.42	16%	0.16	0.02
	≤3 months	3	OG:60/CG:61	−0.78 (−1.06, −0.50)	0.93	5.52	65%	<0.00001	

Notes: *p*^a^, test of combined effect; *p*^b^, comparison between subgroups. BMI: body mass index, 6MWD: six-minute walk distance, Peak VO_2_: maximal rate of oxygen consumption, FVC% pred: predicted forced vital capacity, DLCO% pred: predicted diffusing capacity of the lung for carbon monoxide, TLC% pred: predicted total lung capacity, SGRQ: St. George’s Respiratory Questionnaire total score, mMRC: modified Medical Research Council score. OG: operational group, CG: control group. A–R–F–B: aerobic–resistance–flexibility–breathing exercise, A–R: aerobic–resistance exercise, A–F: aerobic–flexibility exercise, Moderate intensity: 40–59% HRR or %VO_2_R, or 64–76% HRmax, or 46–63% VO_2_max, or fair light to somewhat hard (RPE 12–13), Light intensity: 30–39% HRR or %VO_2_R, or 57–63% HRmax, or 37–45% VO_2_max, or very light–fairly light (RPE 9–11). According to the heterogeneity, the six indexes of 6MWD, peak VO_2,_ FVC% pred, DLCO% pred, TLC% pred, SGRQ and mMRC were analyzed by subgroup. According to the research characteristics of the included studies, we conducted a subgroup analysis based on the exercise frequency, intensity, type, duration, age and BMI. The *d* values of 0.2, 0.5, 0.8 represent small, medium, and large effect sizes.

## Data Availability

The data presented in this study are available on request from the corresponding author.
